# Identifying Anaerobic Bacteria Using MALDI-TOF Mass Spectrometry: A Four-Year Experience

**DOI:** 10.3389/fcimb.2021.521014

**Published:** 2021-04-22

**Authors:** Luis Alcalá, Mercedes Marín, Adrián Ruiz, Lidia Quiroga, Maribel Zamora-Cintas, María Antonia Fernández-Chico, Patricia Muñoz, Belén Rodríguez-Sánchez

**Affiliations:** ^1^ Clinical Microbiology and Infectious Diseases Department, Hospital General Universitario Gregorio Marañón, Madrid, Spain; ^2^ Instituto de Investigación Sanitaria Gregorio Marañón, Madrid, Spain; ^3^ CIBER de Enfermedades Respiratorias (CIBERES CB06/06/0058), Madrid, Spain; ^4^ Medicine Department, School of Medicine, Universidad Complutense de Madrid, Madrid, Spain

**Keywords:** MALDI-TOF, mass spectrometry, protein spectrum, anaerobic bacteria, routine identification

## Abstract

Because of the special culture requirements of anaerobic bacteria, their low growth-rate and the difficulties to isolate them, MALDI-TOF MS has become a reliable identification tool for these microorganisms due to the little amount of bacteria required and the accuracy of MALDI-TOF MS identifications. In this study, the performance of MALDI-TOF MS for the identification of anaerobic isolates during a 4-year period is described. Biomass from colonies grown on Brucella agar was directly smeared onto the MALDI-TOF target plate and submitted to on-plate protein extraction with 1μl of 100% formic acid. Sequencing analysis of the 16S rRNA gene was used as a reference method for the identification of isolates unreliably or not identified by MALDI-TOF MS. Overall, 95.7% of the isolates were identified to the species level using the updated V6 database vs 93.8% with previous databases lacking some anaerobic species; 68.5% of the total were reliably identified with high-confidence score values (≥2.0) and 95.0% with low-confidence values (score value ≥1.7). Besides, no differences between Gram-positive and Gram-negative isolates were detected beyond a slight decrease of correct species assignment for gram positive cocci (94.1% vs 95.7% globally). MALDI-TOF MS has demonstrated its usefulness for the identification of anaerobes, with high correlation with phenotypic and conventional methods. Over the study period, only 2.1% of the isolates could not be reliably identified and required molecular methods for a final identification. Therefore, MALDI-TOF MS provided reliable identification of anaerobic isolates, allowing clinicians to streamline the most appropriate antibiotic therapy and manage patients accordingly.

## Introduction

Over the last decade, MALDI-TOF MS has demonstrated to be a rapid, accurate and inexpensive alternative for the identification of bacteria species encountered in the microbiology laboratory (Croxatto et al., 2012; Dingle and Butler-Wu, 2013; Rodríguez-Sánchez et al., 2014; Patel, 2015). This technology has proved to be highly useful for the identification of anaerobic bacteria since only 2-3 colonies from agar plates are enough to successfully identify the species they belong to, the identification can be obtained in 5-10 minutes and only a few reagents are needed in very small amounts (Nagy et al., 2012; Schmitt et al., 2013; Garner et al., 2014; Lee et al., 2015; Rodríguez-Sánchez et al., 2016; Xiao et al., 2016; Ferrand et al., 2018).

The level of expertise acquired on the implementation of MALDI-TOF for the identification of anaerobic isolates has also enabled their direct identification from blood cultures (Jeverica et al., 2018) and the determination of their antibiotic susceptibility patterns (Nagy et al., 2011; Treviño et al., 2012). The only drawback of MALDI-TOF MS so far has been the lack of identification of species either missing or underrepresented in the available databases. MALDI-TOF users have detected this limitation, especially in the case of Gram positive anaerobic cocci, underrepresented in the available databases (Veloo et al., 2016). A multicenter study has been performed in order to expand and validate new reference spectra (Main Spectral Profiles, MSPs) corresponding to less common anaerobic bacteria. The input from this study has allowed the V6 database from Bruker Daltonics (Bremen, Germany) –containing 6903 MSPs- to increase the number of MSPs from clinically important anaerobic bacteria and to comprise a higher number of anaerobe species (Veloo et al., 2018).

A previous study carried out in our laboratory demonstrated that the implementation of MALDI-TOF MS for the routine identification of anaerobes reduced the number of isolates that required DNA sequencing analysis for a conclusive species assignment to 3.1% (9/295). Besides, correct species-level identification was achieved in 85.8% of the cases and no misidentifications at the genus level were detected (Rodríguez-Sánchez et al., 2016). Since the database available at that time contained 5627 MSPs and was previous to the enrichment with anaerobic reference spectra we hypothesize that the current database could increase the rate of species-level identification of anaerobic species. For that purpose, we analyzed the anaerobic isolates routinely identified in the Hospital Gregorio Marañón (Madrid, Spain) between 2013 and 2016 using MALDI-TOF MS and the V6 database, enriched in anaerobic species. The reference method in our study was the analysis of the 16S rRNA gene sequence, performed on the isolates not reliably identified by MALDI-TOF MS and on those that belonged to species that had not been evaluated in our previous study.

## Material and Methods

### Isolates

During the study period - January 2013 to December 2016-, 4094 anaerobic strains were isolated from clinical samples and subsequently identified in the microbiology laboratory from the Hospital Gregorio Marañón (Madrid, Spain). The isolates belonged to 190 species and 50 genera (**Supplementary Table 1**). None of the isolates within this study had been included in previous articles focusing on the evaluation of MALDI-TOF for the identification of anaerobic bacteria. *Clostridioides difficile* was considered in this study as *Clostridium difficile*, since this is how MALDI-TOF MS currently identifies this microorganism, even with the most upgraded library (9234 MSPs) –Bruker Daltonics-.

All clinical samples –sourced from abscesses (32.7%), soft tissue biopsies (23.2%), wound exudates (12.5%), blood (8.4%), peritoneal fluids (8.2%) and others (15.0%)- were cultured on Brucella agar (Becton Dickinson, NJ, USA) and incubated at 35°C for 48 hours in anaerobic conditions. An aerotolerance test was performed on suspect colonies grown on the agar plates and those confirmed as anaerobic bacteria were submitted to identification by MALDI-TOF MS. Only those isolates unreliably identified by MALDI-TOF MS or belonging to a species not encountered previously in our laboratory (Rodríguez-Sánchez et al., 2016) were further identified by DNA sequencing analysis.

### Conventional and Genomic Identification of the Anaerobic Isolates

Direct microscopic observation of the bacteria grown under anaerobic conditions was performed. Gram staining was also performed when more than one species from the same clinical sample was suspected and for confirmation purposes. Besides, all those isolates whose identification by MALDI-TOF MS was genus-level, not reliable or yielded a species that had not been previously evaluated in our laboratory were further identified by amplification of the 5’ end 16S rRNA gene with the universal primers E8F -5’-AGAGTTTGATCCTGGCTCAG-3’- and E533R -5`-TTACCGCGGCTGCTGGCA-3’- (Baker et al., 2003; Rodríguez-Sánchez et al., 2016). Further details about the amplification conditions, PCR product purification and sequencing have been provided before (Rodríguez-Sánchez et al., 2014). The identification obtained was interpreted following the CLSI guidelines (CLSI, 2008) and considered as the reference identification of the anaerobic isolates included in this study (**Supplementary Table 2**).

### Identification Using MALDI-TOF MS

All anaerobic isolates were analyzed using a Microflex LT bench top mass spectrometer (Bruker Daltonics, Bremen, Germany). FlexControl 3.3 and Maldi Biotyper 3.1 (Bruker Daltonics) were used for the mass spectrometer control and comparison with the database, respectively. The MBT library (Bruker Daltonics) containing 9234 MSPs was used. All spectra acquired before the V6 database was released were re-identified with it for this study.

Sample preparation has been described elsewhere (Rodríguez-Sánchez et al., 2014). Briefly, it consisted on spotting a small amount of bacteria with a 1μl sterile loop or a toothpick onto a MALDI target plate. An on-target protein extraction step was performed by overlaying the sample with 1μl of 100% formic acid and allowing it to dry at room temperature. Once dried, the spots were covered with 1μl of matrix -α-HCCA, prepared according to the manufacturer’s instructions-. When the mixture was dried, spectra acquisition was performed using default settings and compared with the database.

A Bacterial Test Standard provided by the manufacturer was included in every run for calibration purposes. Default settings (acquisition of mass spectra in the linear positive mode within the 2-20kDa range) were applied. All isolates were analyzed by MALDI-TOF MS in duplicates and the higher score value was recorded as well as the identification provided by MALDI-TOF MS.

### Interpretation of the Results

In this study, score values ≥2.0 and ≥1.7 were established as the ranges for high- and low-confidence identification, respectively. A lower cut-off (1.8) for species-level identification was also analyzed. This cut-off has already been applied by other authors (Fedorko et al., 2012; Hsu and Burnham, 2014; Rodríguez-Sánchez et al., 2016). Isolates identified with score values below 1.6 were only taken into account when the first three identifications provided by MALDI-TOF MS were consistent at the species or at the genus level. Otherwise, the identification was considered “not reliable”.

When the analysis of the 16S rRNA gene sequencing was performed, the identifications provided by this method and by MALDI-TOF were considered as 1) concordant at the species level, 2) concordant only at the genus level or 3) discordant.

### Ethics Statement

The Hospital Gregorio Marañón Ethics Committee approved and gave consent for the performance of this study (Code: MALDI-Anaerobios). The study has been carried out using microbiological samples, not human products. Therefore, all the conditions to waive the informed consent have been met.

## Results

### Distribution of the Anaerobic Strains

Among the isolates analyzed, *Bacteroides* was the most commonly encountered genus with 763 isolates included in this study (18.5%); *Propionibacterium* spp. [now *Cutibacterium* spp. (Scholz and Kilian, 2016)] was the second most abundant genus (n=485, 11.8%) followed by *Prevotella* spp. (n=448, 10.9%), *Finegoldia* spp. (n=299, 7.3%) and *Parvimonas* spp. (n=255, 6.2%) (**Table 1**).

**Table 1:** List of anaerobic isolates identified by MALDI-TOF MS.

LIST OF MICROORGANISMS	Number of isolates	MICROORGANISMS IDENTIFIED BY MALDI-TOF (%)						
		Species Level	Genus Level	Not Reliable/No ID	Score ≥2.0	Score 1.99-1.70	Score 1.69-1.60	Score <1.6
**Gram-negative bacilli**								
*Alistipes finegoldii*	1	1	–	–	–	1	–	–
*Alistipes onderdonkii*	5	5	–	–	5	–	–	–
*Bacteroides caccae*	8	8	–	–	7	1	–	–
*Bacteroides fragilis*	359	356	3	–	332	20	2	5
*Bacteroides ovatus*	73	72	1	–	48	20	3	2
*Bacteroides pyogenes*	11	11	–	–	6	4	1	–
*Bacteroides thetaiotaomicron*	152	151	1	–	127	23	2	–
*Bacteroides uniformis*	33	33	–	–	32	1	–	–
*Bacteroides vulgatus*	92	91	1	–	65	25	1	1
*Bacteroides* sp.^1^	32	32	–	–	14	18	–	–
*Bilophila wadsworthia*	3	3	–	–	1	2	–	–
*Bilophila* sp.	3	–	3	–	–	3	–	–
*Butyricimonas virosa*	1	1	–	–	1	–	–	–
***Campylobacter rectus***	2	2	–	–	1	1	–	–
***Campylobacter ureolyticus***	2	2	–	–	1	1	–	–
*Capnocytophaga gingivalis*	3	3	–	–	1	2	–	–
*Capnocytophaga granulosa*	2	2	–	–	2	–	–	–
*Capnocytophaga ochracea*	2	2	–	–	2	–	–	–
*Capnocytophaga sputigena*	4	4	–	–	3	1	–	–
*Capnocytophaga* sp.	3	–	3	–	3	–	–	–
***Dialister micraerophilus***	4	4	–	–	4	–	–	–
***Dialister pneumosintes***	25	25	–	–	25	–	–	–
*Fusobacterium naviforme*	19	17	2	–	6	10	2	1
*Fusobacterium necrophorum*	61	60	1	–	50	10	–	1
*Fusobacterium nucleatum*	135	128	2	5	63	53	7	12
*Fusobacterium periodonticum*	6	6	–	–	–	4	2	–
*Fusobacterium* sp.^2^	16	14	2	–	8	6	2	–
*Odoribacter splanchnicus*	1	1	–	–	1	–	–	–
*Parabacteroides distasonis*	41	41	–	–	41	–	–	–
*Parabacteroides goldsteinii*	6	6	–	–	6	–	–	–
*Parabacteroides johnsonii*	11	11	–	–	1	8	2	–
*Porphyromonas endodontalis*	2	–	–	2	–	–	–	2
*Porphyromonas gingivalis*	1	1	–	–	–	1	–	–
*Porphyromonas somerae*	9	9	–	–	6	2	1	–
*Porphyromonas uenonis*	2	2	–	–	–	–	1	1
*Prevotella baroniae*	26	26	–	–	20	6	–	–
*Prevotella bergensis*	10	10	–	–	5	5	–	–
*Prevotella bivia*	53	53	–	–	41	12	–	–
*Prevotella buccae*	57	56	–	1	45	11	–	1
*Prevotella denticola*	37	36	–	1	30	6	–	1
*Prevotella disiens*	20	19	–	1	11	7	–	2
*Prevotella intermedia*	55	53	–	2	36	17	–	2
*Prevotella melaninogenica*	52	52	–	–	19	26	5	2
*Prevotella nigrescens*	31	31	–	–	24	6	1	–
*Prevotella oris*	20	20	–	–	19	1	–	–
*Prevotella* sp.*^3^*	87	54	17	16	31	30	3	23
	**1578**	**1514 (95.9)**	**36 (2.3)**	**28 (1.8)**	**1143 (72.4)**	**344 (21.8)**	**35 (2.2)**	**56 (3.6)**
**Gram-negative cocci**								
*Acidaminococcus intestini*	8	8	–	–	7	1	–	–
*Megasphaera micronuciformis*	2	2	–	–	2	–	–	–
*Veillonella atypica*	23	23	–	–	21	2	–	–
*Veillonella dispar*	15	14	–	1	10	4	–	1
*Veillonella parvula*	137	137	–	–	124	12	1	–
*Veillonella ratti*	2	2	–	–	1	1	–	–
	**187**	**186 (99.5)**	**0 (0.0)**	**1 (0.5)**	**165 (88.2)**	**20 (10.7)**	**1 (0.5)**	**1 (0.5)**
**Gram-positive bacilli**								
*Actinomyces europaeus*	17	16	–	1	2	14	–	1
*Actinomyces meyeri/odontolyticus*	82	77	1	4	34	41	1	6
*Actinomyces neuii*	17	17	–	–	14	3	–	–
*Actinomyces oris*	15	15	–	–	13	2	–	–
*Actinomyces radingae*	15	14	–	1	11	3	–	1
*Actinomyces turicensis*	31	31	–	–	25	6	–	–
*Actinomyces urogenitalis*	8	8	–	–	8	–	–	–
*Actinomyces* sp.*^4^*	6	6	–	–	3	3	–	–
*Actinotignum schaalii*	24	23	–	1	12	10	1	1
*Alloscardovia omnicolens*	1	1	–	–	–	1	–	–
*Atopobium minutum*	7	7	–	–	6	1	–	–
*Atopobium parvulum*	31	30	–	1	24	6	–	1
*Atopobium rimae*	13	13	–	–	12	1	–	–
*Atopobium vaginae*	5	5	–	–	4	1	–	–
*Bifidobacterium longum*	12	12	–	–	10	2	–	–
*Bifidobacterium* sp.^5^	11	11	–	–	6	5	–	–
*Blautia coccoides*	1	1	–	–	–	1	–	–
*Clostridium clostridioforme*	10	8	–	2	6	1	1	2
*Clostridium difficile*	29	25	–	4	22	1	2	4
*Clostridium innocuum*	37	36	1	–	11	25	1	–
*Clostridium perfringens*	76	73	3	–	70	3	–	3
*Clostridium ramosum*	13	13	–	–	12	1	–	–
*Clostridium* sp.^6^	55	53	–	2	34	16	5	–
*Collinsella aerofaciens*	8	8	–	–	6	2	–	–
*Coprobacillus cateniformis*	1	1	–	–	–	1	–	–
*Eggerthella lenta*	71	66	–	5	61	5	–	5
*Eggerthia catenaformis*	6	6	–	–	3	3	–	–
*Eubacterium brachy*	6	6	–	–	6	–	–	–
*Eubacterium limosum*	3	3	–	–	3	–	–	–
*Eubacterium yurii*	1	1	–	–	–	1	–	–
*Flavonifractor plautii*	6	6	–	–	4	2	–	–
*Hungatella hathewayi*	13	13	–	–	12	1	–	–
*Lachnoanaerobaculum orale*	4	4	–	–	2	2	–	–
*Lachnoanaerobaculum umeaense*	9	9	–	–	–	7	2	–
***Lactobacillus fermentum***	10	8	2	–	2	6	–	2
***Lactobacillus gasseri***	29	29	–	–	28	–	1	–
***Lactobacillus jensenii***	12	11	–	1	7	3	1	1
***Lactobacillus paracasei***	28	26	1	1	24	1	1	2
***Lactobacillus rhamnosus***	51	50	1	–	43	8	–	–
***Lactobacillus* sp.*^7^***	34	33	1	–	23	10	–	1
*Mobiluncus curtisii*	6	3	2	1	–	4	1	1
***Leuconostoc lactis***	1	1	–	–	–	1	–	–
*Olsenella uli*	12	11	–	1	6	5	–	1
*Propionibacterium acidifaciens*	13	13	–	–	6	7	–	–
*Propionibacterium acnes*	409	400	–	9	202	191	5	11
*Propionibacterium avidum*	42	41	–	1	24	17	–	1
*Propionibacterium granulosum*	10	10	–	–	4	6	–	–
*Propionibacterium* sp.^8^	11	1	10	–	7	2	1	1
*Propionimicrobium lymphophilum*	6	6	–	–	–	6	–	–
*Ruminococcus gnavus*	3	3	–	–	1	2	–	–
*Slackia exigua*	43	43	–	–	39	2	1	1
*Trueperella bernardiae*	7	6	1	–	4	2	–	1
*Solobacterium moorei*	35	34	–	1	30	4	–	1
	**1406**	**1347 (95.8)**	**23 (1.6)**	**36 (2.6)**	**886 (63.0)**	**448 (31.9)**	**24 (1.7)**	**48 (3.4)**
**Gram-positive cocci**								
*Anaerococcus hydrogenalis*	19	13	4	2	12	2	2	3
*Anaerococcus murdochii*	15	15	–	–	6	8	1	–
*Anaerococcus vaginalis*	68	66	2	–	8	59	–	1
*Anaerococcus* sp.^9^	32	16	15	1	16	11	1	4
*Finegoldia magna*	299	290	–	9	192	95	3	9
***Gemella haemolysans***	5	5	–	–	3	2	–	–
***Gemella morbillorum***	18	17	–	1	14	3	–	1
***Gemella sanguinis***	5	5	–	–	5	–	–	–
*Helcococcus kunzii*	4	4	–	–	4	–	–	–
*Murdochiella asaccharolytica*	3	3	–	–	3	–	–	–
*Parvimonas micra*	255	253	–	2	233	19	1	2
***Pediococcus pentosaceus***	1	1	–	–	1	–	–	–
*Peptococcus niger*	10	9	–	1	5	3	1	1
*Peptoniphilus gorbachii*	10	9	–	1	1	6	2	1
*Peptoniphilus harei*	126	124	–	2	70	52	–	4
*Peptoniphilus* sp.^10^	17	4	13	–	8	9	–	–
*Peptostreptococcus anaerobius*	36	35	1	–	31	3	1	1
	**923**	**869 (94.1)**	**35 (3.8)**	**19 (2.1)**	**612 (66.3)**	**272 (29.5)**	**12 (1.3)**	**27 (2.9)**
**TOTAL**	**4094**	**3916 (95.7)**	**94 (2.3)**	**84 (2.1)**	**2806 (68.5)**	**1084 (26.5)**	**72 (1.8)**	**132 (3.2)**

Both the level of identification (species-, genus-level or no identification) and the score values provided by the mass spectrometer are stated. Percentages are represented in brackets. Facultative anaerobes are shown in bold. ^1^Bacteroides cellulosilyticus, B. coagulans, B. faecis, B. finegoldii, B. intestinalis, B. massiliensis, B. nordii, B. salyersiae and B. stercoris. ^2^Fusobacterium canifelinum, F. gonidiaformans, F. mortiferum, F. ulcerans, F. varium and Fusarium sp. ^3^Prevotella amnii, P. buccalis, P. corporis, P. dentalis, P. heparinolytica, P. histicola, P. loescheii, P. nanceiensis, P. oralis, P. pallens, P. salivae, P. stercorea, P. timonensis and Prevotella sp. ^4^Actinomyces israelii, A. funkei, A. graevenitzii and A. naeslundii. ^5^Bifidobacterium adolescentis, B. breve, B. catenulatum, B. dentium and B. pseudocatenulatum. ^6^Clostridium aldenense, C. bifermentans, C. bolteae, C. butyricum, C. celerecrescens, C. citroniae, C. colicanis, C. disporicum, C. glycolicum, C. halophilum, C. hylemonae, C. limosum, C. mayambei, C. paraputrificum, C. scindens, C. septicum, C. sordelli, C. sphenoides, C. sporogenes, C. subterminale, C. symbiosum, C. tertium and C. tetani. ^7^Lactobacillus amylovorus, L. casei, L. crispatus, L. curvatus, L. delbruckii, L. iners, L. johnsonii, L. mucosae, L. oris, L. plantarum, L. reuteri, L. salivarius, L. vaginalis and Lactobacillus sp. ^8^Propionibacterium propionicum and Propionibacterium sp. ^9^Anaerococcus lactolyticus, A. octavius, A. prevotii, A. tetradius and Anaerococcus sp. ^10^Peptoniphilus koenoeneniae, P. lacrimalis, P. tyrrelliae and Peptoniphilus sp. B. ovatus/xylanisolvens, B. vulgatus/dorei cannot be differentiated by MALDI-TOF.

### Identification of Anaerobic Strains

The implementation of MALDI-TOF MS for the identification of anaerobic isolates yielded 95.7% (n=3916), 2.3% (n=94) and 2.1% (n=84) species-level, genus-level and unreliable identifications, respectively (**Table 1**). For the last two categories 16S rRNA gene sequencing was needed for species assignment (**Supplementary Table 2**). Besides, 237 isolates identified at the species level by MALDI-TOF MS yielded species that had never been found before in our laboratory and were identified for confirmatory purposes. These isolates belonged mainly to genera *Bacteroides*, *Fusobacterium*, *Prevotella*, *Actinomyces*, *Clostridium*, *Lactobacillus* and *Propionibacterium* (**Supplementary Table 2**).

From the Gram negative microorganisms, 1514/1578 bacilli (95.9%) and 186/187 cocci (99.5%) were identified at the species level. Most of the isolates not reliably identified belonged to the species *Fusobacterium nucleatum* (n=5) and to the genus *Prevotella* (n=15). Overall, 72.4% of the bacilli and 88.2% of the cocci were identified with high-confidence score values (score≥2.0) and with low-confidence values (score from ≥1.7) 21.8% of the bacilli and 10.7% of the cocci (**Table 1**). Besides, 90.0% of the bacilli and 98.4% of the cocci were reliably identified at the species level with score values ≥1.8, a cut-off proposed for high-confidence species-level assignment by different authors - (Fedorko et al., 2012; Hsu and Burnham, 2014; Rodríguez-Sánchez et al., 2016)- (**Supplementary Table 1**).

From the Gram positive microorganisms, 1347/1406 bacilli (95.8%) and 869/923 cocci (94.1%) were identified at the species level. Besides, 23 bacilli (1.6%) and 35 cocci (3.5%) were identified at the genus level. The bacilli belonged mainly to the genera *Clostridium* (n=4), *Lactobacillus* (n=5) and *Propionibacterium* (n=10) and the cocci to the genera *Anaerococcus* (n=21) and *Peptoniphilus* (n=13) –**Table 1**-. Finally, 36 bacilli (2.6%) and 19 cocci (2.1%) could not be reliably identified by MALDI-TOF MS. They belonged mostly to the genera *Actinomyces* (n=6), *Clostridium* (n=8), *Eggerthella* (n=5) and *Propionibacterium* (n=10) in the first case and to *Finegoldia magna* (n=9) in the second case. The lower score values registered lie within this group of unreliably identified isolates (**Table 1**).

According to the cut-off established by the manufacturer, 68.5% of the isolates (2806) were identified with score values ≥2.0 and 26.5% (1084) with score values ≥1.7, accounting for a total of 95.0% reliable identification. From the remaining 5.0%, isolates belonging to commonly encountered species and well represented in the databases such as *Bacteroides fragilis* or *Prevotella melaninogenica*, were reliably identified despite the low score values.

**Figure 1:**
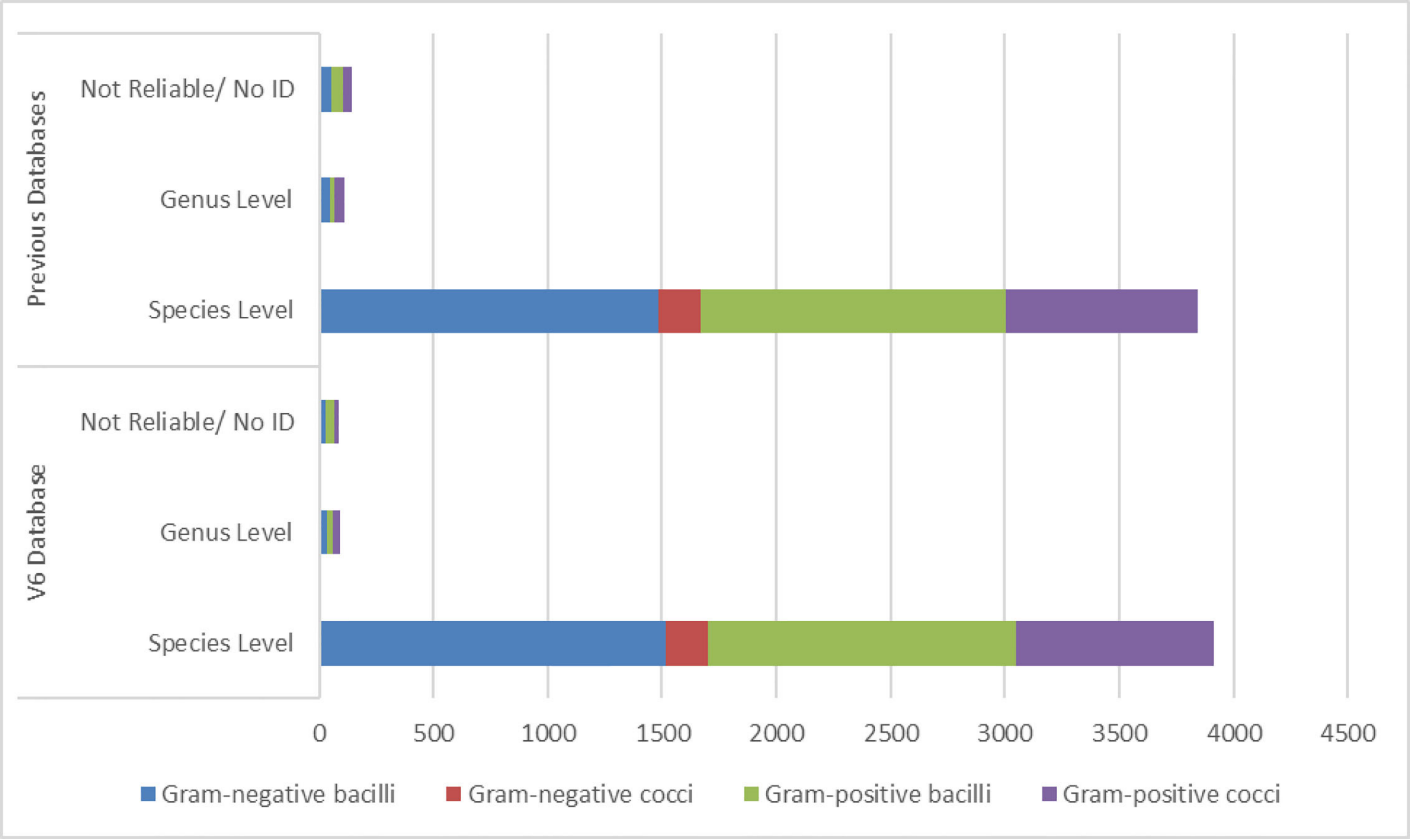
Representation of the species-level (Sp) and genus-level (Gn) identification rates as well as the percentages of not reliable or no identifications (NR/No ID) using the V6 database and previous databases. The V6 database provided 95.9%, 2.3% and 1.8% Sp, Gn and NR/No ID identification for Gram-negative bacilli (in blue) vs 93.9%, 2.8% and 3.2% with previous libraries. For Gram-positive cocci (in red), the rates of Sp (99.5%), Gn (0.0%) and NR/No ID (0.5%) did not change with the different databases. However, for Gram positive bacilli (in green) -Sp 95.8%, Gn 1.6% and NR/No ID 2.6%- and specially for Gram positive cocci (in purple) -Sp 94.1%, Gn 3.8% and NR/No ID 2.1%- the rates of correct identifications improved when the V6 database was implemented in comparison with previous libraries (Sp 94.8%, Gn 1.7% and NR/No ID 3.5% for Gram positive bacilli and Sp 91.4%, Gn 4.3% and NR/No ID 4.2% for Gram positive cocci).

The enrichment of the available databases has made possible the identification of an increasing number of anaerobic isolates. In our study, 70 isolates that could not be previously identified using older databases obtained correct species-assignment when the Biotyper V6 library or a more upgraded database was applied (**Table 2**). The addition of reference spectra from anaerobic isolates to this library allowed the identification at the species level of 56/70 isolates (**Figure 1**). Only 8 isolates belonging to *Prevotella* spp. one *Propionibacterium* spp. and 5 to *Anaerococcus* spp. were identified only at the genus level. Besides, their identification was achieved with score values ≥1.6 in all but 8 cases, but the identification was reliable nonetheless due to the consistency within the top ten identifications provided by MALDI-TOF MS.

**Table 2:** Isolates identified by MALDI-TOF MS only when the Biotyper V6 database –or a more upgraded library- was implemented.

IDENTIFICATION BY VISUAL INSPECTION	IDENTIFICATION WITH BIOTYPER V6 LIBRARY	SCORE
**Gram negative bacilli**	*Bacteroides pyogenes*	1,64
	*Bilophila wadsworthia*	1,82
	*Bilophila wadsworthia*	1,91
	*Bilophila wadsworthia*	2,24
	*Fusobacterium canifelinum*	1,78
	*Fusobacterium nucleatum*	1,61
	*Fusobacterium nucleatum*	1,62
	*Odoribacter splanchnicus*	2,24
	*Parabacteroides goldsteinii*	2,12
	*Porphyromonas somerae*	2,08
	*Porphyromonas somerae*	2,29
	*Porphyromonas somerae*	2,20
	*Porphyromonas somerae*	2,08
	*Porphyromonas somerae*	2,02
	*Porphyromonas uenonis*	1,67
	*Porphyromonas uenonis*	1,52
	*Prevotella heparinolytica*	2,27
	*Prevotella heparinolytica*	2,19
	*Prevotella loescheii*	1,92
	*Prevotella melaninogenica*	1,65
	*Prevotella melaninogenica*	1,59
	*Prevotella nigrescens*	1,66
	*Prevotella* sp.	1,59
	*Prevotella* sp.	1,63
	*Prevotella* sp.	1,65
	*Prevotella* sp.	1,66
	*Prevotella* sp.	1,66
	*Prevotella* sp.	1,69
	*Prevotella* sp.	1,72
	*Prevotella* sp.	1,99
**Gram positive bacilli**	*Actinomyces europaeus*	1,57
	*Clostridium difficile*	1,65
	*Clostridium mayambei*	1,72
	*Lactobacillus jensenii*	1,62
	*Propionibacterium acnes*	1,65
	*Propionibacterium acnes*	1,71
	*Propionibacterium acnes*	1,75
	*Propionibacterium acnes*	1,70
	*Propionibacterium acnes*	1,69
	*Propionibacterium acnes*	1,65
	*Propionibacterium acnes*	1,46
	*Propionibacterium granulosum*	1,84
	*Propionibacterium propionicum*	1,64
	*Propionibacterium* sp.	1,52
**Gram positive cocci**	*Anaerococcus lactolyticus*	1,71
	*Anaerococcus lactolyticus*	1,75
	*Anaerococcus murdochii*	1,69
	*Anaerococcus murdochii*	1,79
	*Anaerococcus murdochii*	1,80
	*Anaerococcus vaginalis*	1,75
	*Anaerococcus vaginalis*	1,90
	*Anaerococcus* sp.	1,84
	*Anaerococcus* sp.	1,97
	*Anaerococcus* sp.	1,98
	*Anaerococcus* sp.	2,07
	*Anaerococcus* sp.	2,08
	*Murdochiella asaccharolytica*	2,29
	*Murdochiella asaccharolytica*	2,18
	*Parvimonas micra*	1,75
	*Parvimonas micra*	1,83
	*Peptococcus niger*	1,62
	*Peptoniphilus gorbachii*	1,66
	*Peptoniphilus gorbachii*	1,71
	*Peptoniphilus gorbachii*	1,76
	*Peptoniphilus harei*	1,49
	*Peptoniphilus koenoeneniae*	2,05
	*Peptoniphilus lacrimalis*	2,26
	*Peptoniphilus lacrimalis*	2,40
	*Peptoniphilus tyrrelliae*	2,00
	*Peptostreptococcus anaerobius*	1,58

## Discussion

The implementation of MALDI-TOF for the routine identification of anaerobic isolates has allowed the rapid and reliable identification of a high number of anaerobic species. This statement has been demonstrated in the present study: from a large number of isolates analyzed (n=4094), 95.7% of them were correctly identified at the species level. Besides, correlation with phenotypic and conventional methods was shown and consistency with DNA sequencing was demonstrated for a limited number of isolates. Although this is one of the limitations of the study, a previous study carried out by our research team showed 85.8% correct species assignment between MALDI-TOF and DNA sequencing for 295 anaerobic isolates (Rodríguez-Sánchez et al., 2016). The increased percentage of species-level identifications can be explained by the enrichment of the available databases with further reference spectra from anaerobic species.

The ENRIA (European Network of Rapid Identification of Anaerobes) project has represented a significant improvement for the identification of anaerobic isolates using MALDI-TOF MS (Veloo et al., 2018). The addition of well-characterized anaerobic isolates from more than 60 different genera allowed the identification of 79.2% of the isolates included in the validation set. The impact of the enriched library on the identification of Gram positive anaerobic isolates at the species-level was also measured: 86.4% using the Biotyper V6 library including the isolates from the ENRIA project versus 69.2% using the previous library version (V5). In our case, the implementation of the Biotyper V6 library allowed the reliable identification of 94.1% of the Gram positive anaerobic cocci from 10 different genera, but failed to identify 19/923 isolates (2.1%). Although the rate of unidentified Gram positive cocci has been reduced to half by implementing the V6 database, these results still pinpoint the need to include further reference spectra from this group of bacteria to future versions of the commercial libraries, but they also render the number of unidentified Gram positive anaerobic cocci similar to other anaerobic groups (1.8% Gram negative bacilli and 2.6% Gram positive bacilli). Thus, this group of bacteria no longer represents a hindrance for MALDI-TOF thanks to the enrichment of the updated libraries with anaerobic isolates. Actually, these rates of unidentified anaerobes represent a realistic number of samples that a routine laboratory can identify by molecular methods without delaying the final identification results or causing unaffordable over-costs.

When anaerobic species are considered globally, correct species assignment of anaerobic species between 70.8% and 91.2% have been reported using different MALDI-TOF MS platforms (Nagy et al., 2012; Schmitt et al., 2013; Garner et al., 2014; Lee et al., 2015; Rodríguez-Sánchez et al., 2016; Xiao et al., 2016; Ferrand et al., 2018). As expected, the lowest rates corresponded to the identification of less common anaerobic species (Ferrand et al., 2018). This fact was also demonstrated in the present study, where infrequent species (e.g. *Prevotella disiens*, *Clostridium subterminale*, *Mobiluncus curtisii*, etc.) could not be identified by MALDI-TOF due to their absence or underrepresentation in the available database. However, other equally infrequent species in our setting were successfully identified (e.g. *Murdochiella asaccharolytica* or *Peptoniphilus lacrimalis*) thanks to the reference spectra included in the most recent databases.

Recent studies have also reported rapid and reliable identification of anaerobic isolates directly from blood cultures (Jeverica et al., 2018; Shannon et al., 2018). Jeverica et al. reported 84.9% correct identifications with score values ≥1.6 from blood cultures spiked with anaerobic isolates using 5% saponin while Shannon et al. demonstrated that short-incubation (4-6 hours) of a few drops of blood culture broths allowed at least genus-level identification in 33.0% of the cases in a small set of samples.

All in all, MALDI-TOF MS provided a high rate of species-level identifications for anaerobic isolates from clinical samples. The rapid and reliable identification of these isolates has provided clinicians with valuable information about the involvement of these microorganisms in important pathologies such as endocarditis or meningitis (Kestler et al., 2017; Kalay et al., 2019). The results from the present study support these statements. In this scenario, the role of MALDI-TOF MS as a reliable tool for the identification of anaerobic bacteria is becoming critical for laboratory personnel and clinicians alike in order to identify these microorganisms in a rapid and reliable way and provide an optimal management of the affected patients.

## Data Availability Statement

The raw data supporting the conclusions of this article will be made available by the authors, without undue reservation, to any qualified researcher.

## Ethics Statement

The Hospital Gregorio Marañón Ethics Committee approved and gave consent for the performance of this study (Code: MALDI-Anaerobios). The study has been carried out using microbiological samples, not human products. Therefore, all the conditions to waive the informed consent have been met.

## Author Contributions

Study design (LA and BR-S). Morphological characterization of the isolates (LA, MZ-C, and MF-C). Identification of the isolates by DNA sequencing (MM). MALDI-TOF identification (AR, LQ, and BR-S) Manuscript writing (BR-S) Manuscript review (LA, MM, PM, and BR-S). All authors contributed to the article and approved the submitted version.

## Funding

This study has been supported by the Miguel Servet Program from the ISCIII-MICINN (CP14/00220) and the project PI18/00997, both belonging to the Health Research Fund (FIS) of the Carlos III Health Institute (ISCIII, Madrid, Spain), partially financed by the by the European Regional Development Fund (FEDER) ‘A way of making Europe.’ LQ has been funded through the grant PEJ16/MED/TL-1507 from the Government of Madrid, Spain (Programa de Garantía Juvenil) and BR-S is a recipient of a Miguel Servet contract supported by the FIS program (MSII19/00002). The funders had no role in the study design, data collection and analysis, decision to publish, or preparation of the manuscript.

## Conflict of Interest

The authors declare that the research was conducted in the absence of any commercial or financial relationships that could be construed as a potential conflict of interest.
