# Corrigendum: Identifying anaerobic bacteria using MALDI-TOF mass spectrometry: a four-year experience

**DOI:** 10.3389/fcimb.2024.1447946

**Published:** 2024-07-04

**Authors:** Luis Alcalá, Mercedes Marín, Adrián Ruiz, Lidia Quiroga, Maribel Zamora-Cintas, María Antonia Fernández-Chico, Patricia Muñoz, Belén Rodríguez-Sánchez

**Affiliations:** ^1^ Clinical Microbiology and Infectious Diseases Department, Hospital General Universitario Gregorio Marañón, Madrid, Spain; ^2^ Instituto de Investigación Sanitaria Gregorio Marañón, Madrid, Spain; ^3^ CIBER de Enfermedades Respiratorias (CIBERES CB06/06/0058), Madrid, Spain; ^4^ Medicine Department, School of Medicine, Universidad Complutense de Madrid, Madrid, Spain

**Keywords:** MALDI-TOF, mass spectrometry, protein spectrum, anaerobic bacteria, routine identification

**Incorrect Funding**

In the published article, there was an error in the Funding statement. This study has been supported by the Miguel Servet Program from the ISCIII-MICINN (CP14/00220) from the Health Research Fund (FIS) of the Carlos III Health Institute (ISCIII, Madrid, Spain) partially financed by the by the European Regional Development Fund (FEDER) ‘A way of making Europe.’ The correct Funding statement appears below.

**Funding**

This study has been supported by the Miguel Servet Program from the ISCIII-MICINN (CP14/00220) and the project PI18/00997, both belonging to the Health Research Fund (FIS) of the Carlos III Health Institute (ISCIII, Madrid, Spain), partially financed by the by the European Regional Development Fund (FEDER) ‘A way of making Europe.’

The authors apologize for this error and state that this does not change the scientific conclusions of the article in any way. The original article has been updated.

