# Supplementary material for: Identifying Anaerobic Bacteria Using MALDI-TOF Mass Spectrometry: A Four-Year Experience
Source: Front Cell Infect Microbiol. 2021 Apr 22;11:521014. doi: 10.3389/fcimb.2021.521014 (PMC8101409; doi:10.3389/fcimb.2021.521014)
Supplement: Supplementary file 1 [file DataSheet_1.docx]

Supplementary Material

Supplementary Table 1. Comprehensive list of the anaerobic isolates analyzed, their level of identification and the score values assigned to them.

| **LIST OF MICROORGANISMS** | **Number of isolates** | **MICROORGANISMS IDENTIFIED BY MALDI-TOF (%)** | | | | | | | | |
| --- | --- | --- | --- | --- | --- | --- | --- | --- | --- | --- |
|  |  | **Species Level** | **Genus Level** | **Not Reliable/ No ID** | **Score ≥2.0** | **Score 1.99-1.90** | **Score 1.89-1.80** | **Score 1.79-1.70** | **Score 1.69-1.60** | **Score <1.6** |
| **Gram-negative bacilli** |  |  |  |  |  |  |  |  |  |  |
| *Alistipes finegoldii* | 1 | 1 | - | - | - | - | 1 | - | - | - |
| *Alistipes onderdonkii* | 5 | 5 | - | - | 5 | - | - | - | - | - |
| *Bacteroides caccae* | 8 | 8 | - | - | 7 | 1 | - | - | - | - |
| *Bacteroides cellulosilyticus* | 1 | 1 | - | - | 1 | - | - | - | - | - |
| *Bacteroides coagulans* | 4 | 4 | - | - | 1 | 2 | 1 | - | - | - |
| *Bacteroides faecis* | 5 | 5 | - | - | 3 | - | 1 | 1 | - | - |
| *Bacteroides finegoldii* | 3 | 3 | - | - | 3 | - | - | - | - | - |
| *Bacteroides fragilis* | 359 | 356 | 3 | - | 332 | 11 | 4 | 5 | 2 | 5 |
| *Bacteroides intestinalis* | 5 | 5 | - | - | - | - | 2 | 3 | - | - |
| *Bacteroides massiliensis* | 1 | 1 | - | - | - | - | - | 1 | - | - |
| *Bacteroides nordii* | 6 | 6 | - | - | - | 6 | - | - | - | - |
| *Bacteroides ovatus* | 73 | 72 | 1 | - | 48 | 7 | 10 | 3 | 3 | 2 |
| *Bacteroides pyogenes* | 11 | 11 | - | - | 6 | 2 | - | 2 | 1 | - |
| *Bacteroides salyersiae* | 3 | 3 | - | - | 3 | - | - | - | - | - |
| *Bacteroides stercoris* | 4 | 4 | - | - | 3 | - | - | 1 | - | - |
| *Bacteroides thetaiotaomicron* | 152 | 151 | 1 | - | 127 | 14 | 6 | 3 | 2 | - |
| *Bacteroides uniformis* | 33 | 33 | - | - | 32 | - | 1 | - | - | - |
| *Bacteroides vulgatus* | 92 | 91 | 1 | - | 65 | 19 | 5 | 1 | 1 | 1 |
| *Bilophila wadsworthia* | 3 | 3 | - | - | 1 | 1 | 1 | - | - | - |
| *Bilophila sp.* | 3 | - | 3 | - | - | 1 | 1 | 1 | - | - |
| *Butyricimonas virosa* | 1 | 1 | - | - | 1 | - | - | - | - | - |
| *Campylobacter rectus* | 2 | 2 | - | - | 1 | - | - | 1 | - | - |
| *Campylobacter ureolyticus* | 2 | 2 | - | - | 1 | - | 1 | - | - | - |
| *Capnocytophaga gingivalis* | 3 | 3 | - | - | 1 | - | 1 | 1 | - | - |
| *Capnocytophaga granulosa* | 2 | 2 | - | - | 2 | - | - | - | - | - |
| *Capnocytophaga ochracea* | 2 | 2 | - | - | 2 | - | - | - | - | - |
| *Capnocytophaga sputigena* | 4 | 4 | - | - | 3 | 1 | - | - | - | - |
| *Capnocytophaga sp.* | 3 | - | 3 | - | 3 | - | - | - | - | - |
| *Dialister micraerophilus* | 4 | 4 | - | - | 4 | - | - | - | - | - |
| *Dialister pneumosintes* | 25 | 25 | - | - | 25 | - | - | - | - | - |
| *Fusobacterium canifelinum* | 5 | 5 | - | - | 2 | 1 | - | 1 | 1 | - |
| *Fusobacterium gonidiaformans* | 5 | 5 | - | - | 3 | - | 1 | - | 1 | - |
| *Fusobacterium mortiferum* | 1 | 1 | - | - | 1 | - | - | - | - | - |
| *Fusobacterium naviforme* | 19 | 17 | 2 | - | 6 | 4 | 4 | 2 | 2 | 1 |
| *Fusobacterium necrophorum* | 61 | 60 | 1 | - | 50 | 6 | 3 | 1 | - | 1 |
| *Fusobacterium nucleatum* | 135 | 128 | 2 | 5 | 63 | 22 | 17 | 14 | 7 | 12 |
| *Fusobacterium periodonticum* | 6 | 6 | - | - | - | 3 | - | 1 | 2 | - |
| *Fusobacterium ulcerans* | 1 | 1 | - | - | - | 1 | - | - | - | - |
| *Fusobacterium varium* | 2 | 2 | - | - | 2 | - | - | - | - | - |
| *Fusobacterium sp.* | 2 | - | 2 | - | - | - | 1 | 1 | - | - |
| *Odoribacter splanchnicus* | 1 | 1 | - | - | 1 | - | - | - | - | - |
| *Parabacteroides distasonis* | 41 | 41 | - | - | 41 | - | - | - | - | - |
| *Parabacteroides goldsteinii* | 6 | 6 | - | - | 6 | - | - | - | - | - |
| *Parabacteroides johnsonii* | 11 | 11 | - | - | 1 | 4 | 2 | 2 | 2 | - |
| *Porphyromonas endodontalis* | 2 | - | - | 2 | - | - | - | - | - | 2 |
| *Porphyromonas gingivalis* | 1 | 1 | - | - | - | 1 | - | - | - | - |
| *Porphyromonas somerae* | 9 | 9 | - | - | 6 | 1 | 1 | - | 1 | - |
| *Porphyromonas uenonis* | 2 | 2 | - | - | - | - | - | - | 1 | 1 |
| *Prevotella amnii* | 4 | 4 | - | - | 4 | - | - | - | - | - |
| *Prevotella baroniae* | 26 | 26 | - | - | 20 | 2 | 2 | 2 | - | - |
| *Prevotella bergensis* | 10 | 10 | - | - | 5 | 3 | 2 | - | - | - |
| *Prevotella bivia* | 53 | 53 | - | - | 41 | 9 | 3 | - | - | - |
| *Prevotella buccae* | 57 | 56 | - | 1 | 45 | 9 | 2 | - | - | 1 |
| *Prevotella buccalis* | 8 | 8 | - | - | 5 | 1 | 2 | - | - | - |
| *Prevotella corporis* | 8 | 8 | - | - | 4 | 2 | 1 | 1 | - | - |
| *Prevotella dentalis* | 1 | 1 | - | - | 1 | - | - | - | - | - |
| *Prevotella denticola* | 37 | 36 | - | 1 | 30 | 3 | 2 | 1 | - | 1 |
| *Prevotella disiens* | 20 | 19 | - | 1 | 11 | 5 | 1 | 1 | - | 2 |
| *Prevotella heparinolytica* | 7 | 7 | - | - | 7 | - | - | - | - | - |
| *Prevotella histicola* | 3 | 3 | - | - | 2 | - | 1 | - | - | - |
| *Prevotella intermedia* | 55 | 53 | - | 2 | 36 | 7 | 2 | 8 | - | 2 |
| *Prevotella loescheii* | 1 | 1 | - | - | - | 1 | - | - | - | - |
| *Prevotella melaninogenica* | 52 | 52 | - | - | 19 | 15 | 7 | 4 | 5 | 2 |
| *Prevotella nanceiensis* | 7 | 7 | - | - | 4 | - | 2 | - | - | 1 |
| *Prevotella nigrescens* | 31 | 31 | - | - | 24 | 2 | 4 | - | 1 | - |
| *Prevotella oralis* | 8 | 8 | - | - | - | 5 | 1 | 1 | 1 | - |
| *Prevotella oris* | 20 | 20 | - | - | 19 | 1 | - | - | - | - |
| *Prevotella pallens* | 2 | 2 | - | - | - | 2 | - | - | - | - |
| *Prevotella salivae* | 3 | 2 | - | 1 | - | - | 1 | 1 | - | 1 |
| *Prevotella stercorea* | 1 | 1 | - | - | - | - | 1 | - | - | - |
| *Prevotella timonensis* | 2 | 2 | - | - | - | 1 | - | 1 | - | - |
| *Prevotella sp.* | 32 | - | 17 | 15 | 4 | 2 | 1 | 2 | 2 | 21 |
|  | **1578** | **1514 (95.9)** | **36 (2.3)** | **28 (1.8)** | **1143 (72.4)** | **178 (11.3)** | **99 (6.3)** | **67 (4.2)** | **35 (2.2)** | **56 (3.6)** |
|  |  |  |  |  |  |  |  |  |  |  |
| **Gram-negative cocci** |  |  |  |  |  |  |  |  |  |  |
| *Acidaminococcus intestini* | 8 | 8 | - | - | 7 | 1 | - | - | - | - |
| *Megasphaera micronuciformis* | 2 | 2 | - | - | 2 | - | - | - | - | - |
| *Veillonella atypica* | 23 | 23 | - | - | 21 | 2 | - | - | - | - |
| *Veillonella dispar* | 15 | 14 | - | 1 | 10 | 3 | 1 | - | - | 1 |
| *Veillonella parvula* | 137 | 137 | - | - | 124 | 9 | 2 | 1 | 1 | - |
| *Veillonella ratti* | 2 | 2 | - | - | 1 | - | 1 | - | - | - |
|  | **187** | **186 (99.5)** | **0 (0.0)** | **1 (0.5)** | **165 (88.2)** | **15 (8.0)** | **4 (2.1)** | **1 (0.5)** | **1 (0.5)** | **1 (0.5)** |
|  |  |  |  |  |  |  |  |  |  |  |
| **Gram-positive bacilli** |  |  |  |  |  |  |  |  |  |  |
| *Actinomyces europaeus* | 17 | 16 | - | 1 | 2 | 2 | 7 | 5 | - | 1 |
| *Actinomyces israelii* | 1 | 1 | - | - | 1 | - | - | - | - | - |
| *Actinomyces funkei* | 1 | 1 | - | - | - | 1 | - | - | - | - |
| *Actinomyces graevenitzii* | 3 | 3 | - | - | 1 | 2 | - | - | - | - |
| *Actinomyces meyeri/odontolyticus* | 82 | 77 | 1 | 4 | 34 | 12 | 15 | 14 | 1 | 6 |
| *Actinomyces naeslundii* | 1 | 1 | - | - | 1 | - | - | - | - | - |
| *Actinomyces neuii* | 17 | 17 | - | - | 14 | 2 | 1 | - | - | - |
| *Actinomyces oris* | 15 | 15 | - | - | 13 | 1 | - | 1 | - | - |
| *Actinomyces radingae* | 15 | 14 | - | 1 | 11 | 2 | 1 | - | - | 1 |
| *Actinomyces turicensis* | 31 | 31 | - | - | 25 | 2 | 4 | - | - | - |
| *Actinomyces urogenitalis* | 8 | 8 | - | - | 8 | - | - | - | - | - |
| *Actinotignum schaalii* | 24 | 23 | - | 1 | 12 | 5 | 3 | 2 | 1 | 1 |
| *Alloscardovia omnicolens* | 1 | 1 | - | - | - | 1 | - | - | - | - |
| *Atopobium minutum* | 7 | 7 | - | - | 6 | 1 | - | - | - | - |
| *Atopobium parvulum* | 31 | 30 | - | 1 | 24 | 6 | - | - | - | 1 |
| *Atopobium rimae* | 13 | 13 | - | - | 12 | 1 | - | - | - | - |
| *Atopobium vaginae* | 5 | 5 | - | - | 4 | 1 | - | - | - | - |
| *Bifidobacterium adolescentis* | 2 | 2 | - | - | 1 | - | - | 1 | - | - |
| *Bifidobacterium breve* | 3 | 3 | - | - | 1 | 1 | 1 | - | - | - |
| *Bifidobacterium catenulatum* | 1 | 1 | - | - | 1 | - | - | - | - | - |
| *Bifidobacterium dentium* | 3 | 3 | - | - | 2 | 1 | - | - | - | - |
| *Bifidobacterium longum* | 12 | 12 | - | - | 10 | 1 | 1 | - | - | - |
| *Bifidobacterium pseudocatenulatum* | 2 | 2 | - | - | 1 | 1 | - | - | - | - |
| *Blautia coccoides* | 1 | 1 | - | - | - | 1 | - | - | - | - |
| *Clostridium aldenense* | 1 | 1 | - | - | 1 | - | - | - | - | - |
| *Clostridium bifermentans* | 3 | 3 | - | - | 1 | 2 | - | - | - | - |
| *Clostridium bolteae* | 2 | 2 | - | - | 2 | - | - | - | - | - |
| *Clostridium butyricum* | 5 | 5 | - | - | 4 | 1 | - | - | - | - |
| *Clostridium celerecrescens* | 5 | 5 | - | - | 1 | 3 | 1 | - | - | - |
| *Clostridium citroniae* | 1 | 1 | - | - | - | 1 | - | - | - | - |
| *Clostridium clostridioforme* | 10 | 8 | - | 2 | 6 | - | - | 1 | 1 | 2 |
| *Clostridium colicanis* | 1 | 1 | - | - | - | 1 | - | - | - | - |
| *Clostridium difficile* | 29 | 25 | - | 4 | 22 | 1 | - | - | 2 | 4 |
| *Clostridium disporicum* | 1 | 1 | - | - | - | - | 1 | - | - | - |
| *Clostridium glycolicum* | 1 | 1 | - | - | 1 | - | - | - | - | - |
| *Clostridium halophilum* | 4 | 4 | - | - | 1 | 1 | - | 1 | 1 | - |
| *Clostridium hylemonae* | 1 | 1 | - | - | 1 | - | - | - | - | - |
| *Clostridium innocuum* | 37 | 36 | 1 | - | 11 | 18 | 5 | 2 | 1 | - |
| *Clostridium limosum* | 2 | 2 | - | - | 2 | - | - | - | - | - |
| *Clostridium mayambei* | 1 | 1 | - | - | - | - | - | 1 | - | - |
| *Clostridium paraputrificum* | 3 | 3 | - | - | 3 | - | - | - | - | - |
| *Clostridium perfringens* | 76 | 73 | 3 | - | 70 | 2 | 1 | - | - | 3 |
| *Clostridium ramosum* | 13 | 13 | - | - | 12 | 1 | - | - | - | - |
| *Clostridium scindens* | 4 | 4 | - | - | 4 | - | - | - | - | - |
| *Clostridium septicum* | 1 | 1 | - | - | 1 | - | - | - | - | - |
| *Clostridium sordellii* | 3 | 3 | - | - | 3 | - | - | - | - | - |
| *Clostridium sphenoides* | 2 | 2 | - | - | 2 | - | - | - | - | - |
| *Clostridium sporogenes* | 6 | 5 | - | 1 | 2 | 3 | - | - | 1 | - |
| *Clostridium subterminale* | 1 | - | - | 1 | - | - | - | - | 1 | - |
| *Clostridium symbiosum* | 1 | 1 | - | - | 1 | - | - | - | - | - |
| *Clostridium tertium* | 4 | 4 | - | - | 4 | - | - | - | - | - |
| *Clostridium tetani* | 2 | 2 | - | - | - | - | - | - | 2 | - |
| *Collinsella aerofaciens* | 8 | 8 | - | - | 6 | 1 | 1 | - | - | - |
| *Coprobacillus cateniformis* | 1 | 1 | - | - | - | - | 1 | - | - | - |
| *Eggerthella lenta* | 71 | 66 | - | 5 | 61 | 4 | 1 | - | - | 5 |
| *Eggerthia catenaformis* | 6 | 6 | - | - | 3 | 2 | 1 | - | - | - |
| *Eubacterium brachy* | 6 | 6 | - | - | 6 | - | - | - | - | - |
| *Eubacterium limosum* | 3 | 3 | - | - | 3 | - | - | - | - | - |
| *Eubacterium yurii* | 1 | 1 | - | - | - | - | 1 | - | - | - |
| *Flavonifractor plautii* | 6 | 6 | - | - | 4 | 2 | - | - | - | - |
| *Hungatella hathewayi* | 13 | 13 | - | - | 12 | - | 1 | - | - | - |
| *Lachnoanaerobaculum orale* | 4 | 4 | - | - | 2 | 2 | - | - | - | - |
| *Lachnoanaerobaculum umeaense* | 9 | 9 | - | - | - | 2 | 2 | 3 | 2 | - |
| *Lactobacillus amylovorus* | 1 | 1 | - | - | 1 | - | - | - | - | - |
| *Lactobacillus casei* | 8 | 7 | 1 | - | 5 | 1 | - | 1 | - | 1 |
| *Lactobacillus crispatus* | 5 | 5 | - | - | 3 | - | - | 2 | - | - |
| *Lactobacillus curvatus* | 1 | 1 | - | - | - | 1 | - | - | - | - |
| *Lactobacillus delbruckii* | 1 | 1 | - | - | - | 1 | - | - | - | - |
| *Lactobacillus fermentum* | 10 | 8 | 2 | - | 2 | 2 | 2 | 2 | - | 2 |
| *Lactobacillus gasseri* | 29 | 29 | - | - | 28 | - | - | - | 1 | - |
| *Lactobacillus iners* | 3 | 3 | - | - | 2 | 1 | - | - | - | - |
| *Lactobacillus jensenii* | 12 | 11 | - | 1 | 7 | 1 | 1 | 1 | 1 | 1 |
| *Lactobacillus johnsonii* | 3 | 3 | - | - | 3 | - | - | - | - | - |
| *Lactobacillus mucosae* | 2 | 2 | - | - | 1 | - | 1 | - | - | - |
| *Lactobacillus oris* | 3 | 3 | - | - | 2 | - | - | 1 | - | - |
| *Lactobacillus paracasei* | 28 | 26 | 1 | 1 | 24 | - | - | 1 | 1 | 2 |
| *Lactobacillus plantarum* | 2 | 2 | - | - | 2 | - | - | - | - | - |
| *Lactobacillus reuteri* | 2 | 2 | - | - | 2 | - | - | - | - | - |
| *Lactobacillus rhamnosus* | 51 | 50 | 1 | - | 43 | 8 | - | - | - | - |
| *Lactobacillus salivarius* | 1 | 1 | - | - | 1 | - | - | - | - | - |
| *Lactobacillus vaginalis* | 2 | 2 | - | - | 1 | - | 1 | - | - | - |
| *Mobiluncus curtisii* | 6 | 3 | 2 | 1 | - | 1 | 1 | 2 | 1 | 1 |
| *Leuconostoc lactis* | 1 | 1 | - | - | - | - | 1 | - | - | - |
| *Olsenella uli* | 12 | 11 | - | 1 | 6 | 1 | 3 | 1 | - | 1 |
| *Propionibacterium acidifaciens* | 13 | 13 | - | - | 6 | 2 | 3 | 2 | - | - |
| *Propionibacterium acnes* | 409 | 400 | - | 9 | 202 | 90 | 52 | 49 | 5 | 11 |
| *Propionibacterium avidum* | 42 | 41 | - | 1 | 24 | 8 | 7 | 2 | - | 1 |
| *Propionibacterium granulosum* | 10 | 10 | - | - | 4 | 5 | 1 | - | - | - |
| *Propionibacterium propionicum* | 1 | 1 | - | - | - | - | - | - | 1 | - |
| *Propionibacterium sp.* | 10 | - | 10 | - | 7 | - | 1 | 1 | - | 1 |
| *Propionimicrobium lymphophilum* | 6 | 6 | - | - | - | 1 | 3 | 2 | - | - |
| *Ruminococcus gnavus* | 3 | 3 | - | - | 1 | - | 2 | - | - | - |
| *Slackia exigua* | 43 | 43 | - | - | 39 | 1 | 1 | - | 1 | 1 |
| *Trueperella bernardiae* | 7 | 6 | 1 | - | 4 | 2 | - | - | - | 1 |
| *Solobacterium moorei* | 35 | 34 | - | 1 | 30 | 1 | 2 | 1 | - | 1 |
|  | **1406** | **1347 (95.8)** | **23 (1.6)** | **36 (2.6)** | **886 (63.0)** | **218 (15.5)** | **131 (9.3)** | **99 (7.1)** | **24 (1.7)** | **48 (3.4)** |
|  |  |  |  |  |  |  |  |  |  |  |
| **Gram-positive cocci** |  |  |  |  |  |  |  |  |  |  |
| *Anaerococcus hydrogenalis* | 19 | 13 | 4 | 2 | 12 | - | - | 2 | 2 | 3 |
| *Anaerococcus lactolyticus* | 5 | 5 | - | - | 2 | - | 1 | 2 | - | - |
| *Anaerococcus murdochii* | 15 | 15 | - | - | 6 | 3 | 4 | 1 | 1 | - |
| *Anaerococcus octavius* | 2 | 2 | - | - | - | 1 | - | 1 | - | - |
| *Anaerococcus prevotii* | 5 | 4 | - | 1 | - | - | 1 | - | 1 | 3 |
| *Anaerococcus tetradius* | 5 | 5 | - | - | 2 | 1 | 1 | - | - | 1 |
| *Anaerococcus vaginalis* | 68 | 66 | 2 | - | 8 | 20 | 30 | 9 | - | 1 |
| *Anaerococcus sp.* | 15 | - | 15 | - | 12 | 2 | 1 | - | - | - |
| *Finegoldia magna* | 299 | 290 | - | 9 | 192 | 50 | 34 | 11 | 3 | 9 |
| *Gemella haemolysans* | 5 | 5 | - | - | 3 | - | 2 | - | - | - |
| *Gemella morbillorum* | 18 | 17 | - | 1 | 14 | 3 | - | - | - | 1 |
| *Gemella sanguinis* | 5 | 5 | - | - | 5 | - | - | - | - | - |
| *Helcococcus kunzii* | 4 | 4 | - | - | 4 | - | - | - | - | - |
| *Murdochiella asaccharolytica* | 3 | 3 | - | - | 3 | - | - | - | - | - |
| *Parvimonas micra* | 255 | 253 | - | 2 | 233 | 7 | 10 | 2 | 1 | 2 |
| *Pediococcus pentosaceus* | 1 | 1 | - | - | 1 | - | - | - | - | - |
| *Peptococcus niger* | 10 | 9 | - | 1 | 5 | 2 | 1 | - | 1 | 1 |
| *Peptoniphilus gorbachii* | 10 | 9 | - | 1 | 1 | - | - | 6 | 2 | 1 |
| *Peptoniphilus harei* | 126 | 124 | - | 2 | 70 | 32 | 16 | 4 | - | 4 |
| *Peptoniphilus koenoeneniae* | 1 | 1 | - | - | 1 | - | - | - | - | - |
| *Peptoniphilus lacrimalis* | 2 | 2 | - | - | 2 | - | - | - | - | - |
| *Peptoniphilus tyrrelliae* | 1 | 1 | - | - | 1 | - | - | - | - | - |
| *Peptoniphilus sp.* | 13 | - | 13 | - | 4 | 5 | 2 | 2 | - | - |
| *Peptostreptococcus anaerobius* | 36 | 35 | 1 | - | 31 | - | 3 | - | 1 | 1 |
|  | **923** | **869 (94.1)** | **35 (3.8)** | **19 (2.1)** | **612 (66.3)** | **126 (13.6)** | **106 (11.5)** | **40 (4.3)** | **12 (1.3)** | **27 (2.9)** |
| **TOTAL** | **4094** | **3916 (95.7)** | **94 (2.3)** | **84 (2.1)** | **2806 (68.5)** | **537 (13.1)** | **340 (8.3)** | **207 (5.1)** | **72 (1.8)** | **132 (3.2)** |

**Supplementary Table 2.** List of anaerobic isolates identified by 16S rRNA gene sequencing due to their unreliable identification by MALDI-TOF MS or for confirmation reasons in case of uncommon or unevaluated species.

| **LIST OF MICROORGANISMS IDENTIFIED BY 16S rRNA GENE SEQUENCING** | **Number of isolates** |
| --- | --- |
|  |  |
| **Gram-negative bacilli** |  |
| *Alistipes finegoldii* | 1 |
| *Alistipes onderdonkii* | 2 |
| *Bacteroides cellulosilyticus* | 1 |
| *Bacteroides coagulans* | 2 |
| *Bacteroides finegoldii* | 3 |
| *Bacteroides fragilis* | 3 |
| *Bacteroides intestinalis* | 2 |
| *Bacteroides massiliensis* | 1 |
| *Bacteroides nordii* | 2 |
| *Bacteroides ovatus* | 1 |
| *Bacteroides pyogenes* | 1 |
| *Bacteroides salyersiae* | 3 |
| *Bacteroides stercoris* | 3 |
| *Bacteroides thetaiotaomicron* | 1 |
| *Bacteroides vulgatus* | 1 |
| *Bilophila wadsworthia* | 3 |
| *Bilophila sp.* | 1 |
| *Butyricimonas virosa* | 1 |
| *Campylobacter rectus* | 2 |
| *Campylobacter ureolyticus* | 2 |
| *Capnocytophaga gingivalis* | 3 |
| *Capnocytophaga granulosa* | 2 |
| *Capnocytophaga ochracea* | 2 |
| *Capnocytophaga sp.* | 3 |
| *Dialister micraerophilus* | 2 |
| *Dialister pneumosintes* | 3 |
| *Fusobacterium canifelinum* | 2 |
| *Fusobacterium mortiferum* | 1 |
| *Fusobacterium naviforme* | 2 |
| *Fusobacterium necrophorum* | 1 |
| *Fusobacterium nucleatum* | 7 |
| *Fusobacterium periodonticum* | 2 |
| *Fusobacterium ulcerans* | 1 |
| *Fusobacterium varium* | 2 |
| *Fusobacterium sp.* | 2 |
| *Odoribacter splanchnicus* | 1 |
| *Parabacteroides goldsteinii* | 1 |
| *Porphyromonas endodontalis* | 2 |
| *Porphyromonas gingivalis* | 1 |
| *Porphyromonas somerae* | 4 |
| *Porphyromonas uenonis* | 2 |
| *Prevotella amnii* | 2 |
| *Prevotella buccae* | 1 |
| *Prevotella buccalis* | 2 |
| *Prevotella corporis* | 1 |
| *Prevotella dentalis* | 1 |
| *Prevotella denticola* | 1 |
| *Prevotella disiens* | 2 |
| *Prevotella heparinolytica* | 2 |
| *Prevotella histicola* | 2 |
| *Prevotella intermedia* | 2 |
| *Prevotella loescheii* | 1 |
| *Prevotella melaninogenica* | 2 |
| *Prevotella nigrescens* | 1 |
| *Prevotella nanceiensis* | 2 |
| *Prevotella pallens* | 2 |
| *Prevotella salivae* | 1 |
| *Prevotella stercorea* | 1 |
| *Prevotella timonensis* | 2 |
| *Prevotella sp.* | 32 |
| **Gram-negative cocci** |  |
| *Acidaminococcus intestini* | 2 |
| *Megasphaera micronuciformis* | 2 |
| *Veillonella dispar* | 3 |
| *Veillonella ratti* | 2 |
| **Gram-positive bacilli** |  |
| *Actinomyces europaeus* | 2 |
| *Actinomyces israelii* | 1 |
| *Actinomyces funkei* | 1 |
| *Actinomyces graevenitzii* | 3 |
| *Actinomyces meyeri/odontolyticus* | 6 |
| *Actinomyces naeslundii* | 1 |
| *Actinomyces radingae* | 2 |
| *Actinomyces urogenitalis* | 2 |
| *Actinotignum schaalii* | 1 |
| *Alloscardovia omnicolens* | 1 |
| *Atopobium parvulum* | 1 |
| *Atopobium vaginae* | 1 |
| *Bifidobacterium adolescentis* | 2 |
| *Bifidobacterium catenulatum* | 1 |
| *Bifidobacterium dentium* | 3 |
| *Bifidobacterium longum* | 1 |
| *Bifidobacterium pseudocatenulatum* | 2 |
| *Blautia coccoides* | 1 |
| *Clostridium aldenense* | 1 |
| *Clostridium bifermentans* | 3 |
| *Clostridium bolteae* | 2 |
| *Clostridium butyricum* | 2 |
| *Clostridium celerecrescens* | 3 |
| *Clostridium citroniae* | 1 |
| *Clostridium clostridioforme* | 2 |
| *Clostridium colicanis* | 1 |
| *Clostridium difficile* | 4 |
| *Clostridium disporicum* | 1 |
| *Clostridium glycolicum* | 1 |
| *Clostridium halophilum* | 4 |
| *Clostridium hylemonae* | 1 |
| *Clostridium innocuum* | 1 |
| *Clostridium limosum* | 2 |
| *Clostridium mayambei* | 1 |
| *Clostridium paraputrificum* | 2 |
| *Clostridium perfringens* | 3 |
| *Clostridium scindens* | 1 |
| *Clostridium septicum* | 1 |
| *Clostridium sordellii* | 2 |
| *Clostridium sporogenes* | 1 |
| *Clostridium subterminale* | 1 |
| *Clostridium symbiosum* | 1 |
| *Clostridium tertium* | 2 |
| *Clostridium tetani* | 2 |
| *Collinsella aerofaciens* | 1 |
| *Coprobacillus cateniformis* | 1 |
| *Eggerthella lenta* | 5 |
| *Eggerthia catenaformis* | 2 |
| *Eubacterium brachy* | 3 |
| *Eubacterium limosum* | 3 |
| *Eubacterium yurii* | 1 |
| *Flavonifractor plautii* | 2 |
| *Lachnoanaerobaculum orale* | 2 |
| *Lachnoanaerobaculum umeaense* | 2 |
| *Lactobacillus amylovorus* | 1 |
| *Lactobacillus casei* | 1 |
| *Lactobacillus curvatus* | 1 |
| *Lactobacillus delbruckii* | 1 |
| *Lactobacillus fermentum* | 2 |
| *Lactobacillus iners* | 3 |
| *Lactobacillus jensenii* | 2 |
| *Lactobacillus johnsonii* | 3 |
| *Lactobacillus mucosae* | 2 |
| *Lactobacillus oris* | 3 |
| *Lactobacillus paracasei* | 2 |
| *Lactobacillus plantarum* | 2 |
| *Lactobacillus rhamosus* | 1 |
| *Lactobacillus salivarius* | 1 |
| *Mobiluncus curtisii* | 3 |
| *Leuconostoc lactis* | 1 |
| *Olsenella uli* | 2 |
| *Propionibacterium acidifaciens* | 2 |
| *Propionibacterium acnes* | 9 |
| *Propionibacterium avidum* | 2 |
| *Propionibacterium granulosum* | 2 |
| *Propionibacterium propionicum* | 1 |
| *Propionibacterium sp.* | 10 |
| *Propionimicrobium lymphophilum* | 1 |
| *Ruminococcus gnavus* | 3 |
| *Trueperella bernardiae* | 2 |
|  |  |
| **Gram-positive cocci** |  |
| *Anaerococcus hydrogenalis* | 7 |
| *Anaerococcus lactolyticus* | 1 |
| *Anaerococcus murdochii* | 3 |
| *Anaerococcus prevotii* | 3 |
| *Anaerococcus tetradius* | 2 |
| *Anaerococcus vaginalis* | 2 |
| *Anaerococcus sp.* | 15 |
| *Finegoldia magna* | 12 |
| *Gemella haemolysans* | 5 |
| *Gemella morbillorum* | 3 |
| *Gemella sanguinis* | 5 |
| *Helcococcus kunzii* | 4 |
| *Murdochiella asaccharolytica* | 3 |
| *Parvimonas micra* | 5 |
| *Pediococcus pentosaceus* | 1 |
| *Peptococcus niger* | 3 |
| *Peptoniphilus gorbachii* | 3 |
| *Peptoniphilus harei* | 4 |
| *Peptoniphilus koenoeneniae* | 1 |
| *Peptoniphilus lacrimalis* | 2 |
| *Peptoniphilus tyrrelliae* | 1 |
| *Peptoniphilus sp.* | 13 |
| *Peptostreptococcus anaerobius* | 3 |
